# An Update on PYRIN Domain-Containing Pattern Recognition Receptors: From Immunity to Pathology

**DOI:** 10.3389/fimmu.2013.00440

**Published:** 2013-12-09

**Authors:** Rojo A. Ratsimandresy, Andrea Dorfleutner, Christian Stehlik

**Affiliations:** ^1^Division of Rheumatology, Department of Medicine, Feinberg School of Medicine, Northwestern University, Chicago, IL, USA; ^2^Robert H. Lurie Comprehensive Cancer Center, Interdepartmental Immunobiology Center and Skin Disease Research Center, Feinberg School of Medicine, Northwestern University, Chicago, IL, USA

**Keywords:** PYRIN domain, innate immunity, pattern recognition receptor, Nod-like receptor, NLR, AIM2-like receptor, ALR, inflammasome

## Abstract

Cytosolic pattern recognition receptors (PRRs) sense a wide range of endogenous danger-associated molecular patterns as well as exogenous pathogen-associated molecular patterns. In particular, Nod-like receptors containing a pyrin domain (PYD), called NLRPs, and AIM2-like receptors (ALRs) have been shown to play a critical role in host defense by facilitating clearance of pathogens and maintaining a healthy gut microflora. NLRPs and ALRs both encode a PYD, which is crucial for relaying signals that result in an efficient innate immune response through activation of several key innate immune signaling pathways. However, mutations in these PRRs have been linked to the development of auto-inflammatory and autoimmune diseases. In addition, they have been implicated in metabolic diseases. In this review, we summarize the function of PYD-containing NLRPs and ALRs and address their contribution to innate immunity, host defense, and immune-linked diseases.

## Introduction

The innate immune system relies on germline-encoded pattern recognition receptors (PRRs) to detect threats against tissue homeostasis. In response to pathogen infection, tissue damage or environmental stress, inflammatory mediators including cytokines, type I interferons (IFNs), and anti-microbial factors are produced. While Toll-like receptors (TLRs) utilize their TIR domain and RIG-I-like receptors (RLRs) and NLRCs their CARD for downstream signaling upon activation, NLRPs and AIM2-like receptors (ALRs) recruit signaling adaptors through their PYRIN domain (PYD). Active NLRPs and ALRs trigger multiple innate immune effector pathways, but by far the best established function of these PYD-containing proteins is the assembly of inflammasomes, which are large multiprotein platforms that form in response to infection and tissue damage and are responsible for the activation of inflammatory caspases, in particular caspase-1 (1, 2). Thus, a necessity of these PRRs is to be able to promote the clustering of inflammasome adaptors, which is essential for induced proximity-mediated activation of caspase-1 (3). Active caspases then induce inflammatory cell death (pyroptosis), maturation, and/or secretion of the leaderless pro-inflammatory cytokines IL-1β and IL-18, and contribute to the release of the related IL-1α (4, 5) as well as the stress-associated danger signal HMGB1 (6, 7). Furthermore, there is increasing evidence for a broader contribution of inflammasomes to unconventional protein secretion (8), to lipid biogenesis and to the release of inflammatory lipids (9–11). Although not as well-established and in many cases derived from overexpression studies, these proteins have also been linked to transcriptional responses, through activation of NF-κB, IRFs, and MAPKs to regulate pro-inflammatory and anti-microbial gene expression, autophagy, and to affect adaptive immune responses.

### PYRIN domain

The PYD, also referred to as PAAD or DAPIN, is a protein binding domain belonging to the death domain superfamily (12). The structure of several PYDs has been determined, which revealed a bundle of 5- to 6-α-helices. PYDs display distinct negatively and positively charged surface patches, which are indicative of electrostatic interactions to occur during PYD-PYD interactions, reminiscent to other death domain folds (13–18). NLRPs and ALRs both encode an N-terminal PYD, but while NLRPs are further composed of a central nucleotide binding NACHT domain and varying copies of C-terminal leucine-rich repeats, ALRs rather contain one or two copies of the oligonucleotide binding HIN-200 domain at the C-terminus. The PYD is the effector domain required for downstream signaling, while evidence supports a role of the LRR and HIN-200 domain in ligand recognition (19–21). The current model for both PRR families is that ligand recognition promotes a conformational change (15, 21–23), which allows nucleotide binding by the NACHT domain and consequently, enables NLRP oligomerization (24–27), while ALRs cluster alongside the DNA staircase (21). Ultimately, this exposes the PYD in NLRPs and ALRs, thus enabling the recruitment of ASC by homotypic PYD–PYD interactions and clustering of ASC. In the context of inflammasomes, the recruitment and clustering of ASC then triggers its interaction with pro-caspases-1 (3, 28) and -8 (29, 30) and their activation by induced proximity. The precise order of events is still elusive and a recent model proposed spontaneous self-oligomerization of the ASC-PYD, which subsequently facilitates its interaction with NLRP3 and potentially also other PYD-containing PRRs (31). Hence, this model suggests that PYDs contain a dual binding interface (31). The influence of NLRPs on other signaling pathways is even less well understood, but might also occur through these adaptors (32, 33). In contrast to ASC-mediated inflammasome activation in response to KSHV (34), the ALR IFI16 promotes induction of IFN-β through connecting to the common pathway leading to IRF-3 phosphorylation through the adaptor STING (stimulator of IFN genes) (35).

**Figure 1:**
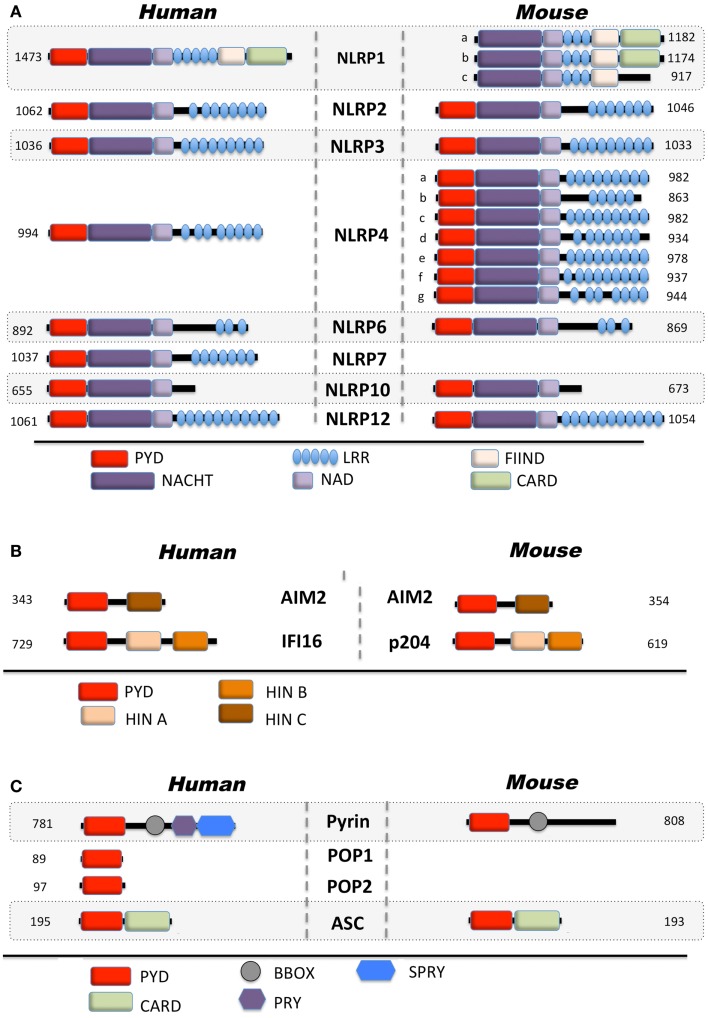
**Domain architecture of PYD-containing proteins involved in innate immunity**. Depicted are human and mouse **(A)** Nod-like receptors, **(B)** AIM2-like receptors, and **(C)** regulatory proteins.

Only 14 NLRPs and 4 ALRs are encoded in humans, while both families are amplified to 34 and 13 members, respectively, in mice (Figures 1A,B). However, the precise function of most family members is still unknown (36–39). Besides NLRPs and ALRs, the PYD is also present in the inflammasome adaptor protein ASC, the regulatory PYD-only proteins (POPs) and Pyrin (Figure 1C) (12). Below, we will specifically discuss the mechanism of activation and function of NLRPs and ALRs, and how defects within these proteins are involved in immune-related disorders.

## Nod-Like Receptors

### NLRP1

NLRP1 (Figure 1A) is also known as NALP1, NAC, DEFCAP, CARD7, and CLR17.1 and has initially been linked to caspase-9 activation within the apoptosome (40).

Inflammasomes were first discovered in 2002 with the initial observation that NLRP1 is able to assemble in an ASC, caspase-1, and caspase-5-containing large inducible protein complex responsible for the autocatalytic activation of caspase-1 in THP-1 cells (1). However, caspase-5 is not recruited to other inflammasomes (25, 41), which is likely due to the unique domain structure of NLRP1. In addition to the common tripartite domain organization of NLRPs, NLRP1 also encodes a C-terminal function to find (FIIND) domain and a CARD, which enables direct caspase-5 recruitment (Figure 1A). Despite its early identification, the *in vivo* function of NLRP1 however remains largely elusive, at least partially due to several key differences between mice and human, which limits the relevance of *in vivo* mouse models. In contrast to human NLRP1, mouse NLRP1 lacks the PYD and exists in three tandem paralog genes (Nlrp1a, Nlrp1b, and Nlrp1c) (Figure 1A). While the PYD is crucial for the recruitment of ASC and subsequently of caspase-1, the C-terminal CARD directly recruits caspase-5, which is necessary for full caspase-1 activation in human cells (1). However, analysis of the first *in vitro*-reconstituted inflammasome with purified recombinant human proteins demonstrated that the core inflammasome components NLRP1 and caspase-1 are sufficient for promoting caspase-1 activation in the presence of NTPs and MDP as a specific agonist (25). In this context, ASC was not necessary, but addition of ASC increased the efficiency of caspase-1 activation. Similar results have also been observed *in vivo* for murine NLRP1b (22). In contrast, a recent analysis suggested that caspase-1 is directly recruited to the C-terminal CARD of NLRP1 and that the PYD is dispensable for inflammasome activation (42). This model could therefore explain NLRP1 inflammasome activation of human and mouse NLRP1, in spite of mouse NLRP1 lacking the PYD. Although the role of the PYD in human NLRP1 is still elusive, the presence of ASC, facilitated by PYD–PYD interaction, could enable an increase in NLRP1-mediated caspase-1 activation in addition to CARD mediated caspase-1/5 recruitment. Additional insights into the molecular mechanism of NLRP1 inflammasome activation came from studies showing that the FIIND domain resembles the autoproteolytic ZU5-like domain found in PIDD, which contains a LRR and a death domain and is part of the caspase-2-activating PIDDosome (43). Accordingly, the FIIND domain in NLRP1 also undergoes autoproteolytic cleavage, which is required for inflammasome activation and congruently, NLRP1b^V988D^, which disrupts the protein conformation required for autoproteolysis, or NLRP1b^S984A^, which disrupts the catalytic serine residue, results in deficient caspase-1 activation without impairing NLRP1 oligomerization (42–44). This step is further regulated by splicing, since an alternative transcript lacking exon 14, which contains the FIIND cleavage site, is deficient in autoproteolytic processing (42). Moreover, rat NLRP1 activation by the *Bacillus anthracis* virulence factor lethal toxin (LTx), a metalloproteinase composed of the pore-forming antigen (PA) and a lethal factor (LF), also cleaves NLRP1, but within the N-terminal domain, suggesting that NLRP1 potentially has several protease cleavage sites (45, 46). Both steps appear necessary for caspase-1 activation, and a possibility could be that the FIIND has partial autoproteolytic activity, and cleavage of NLRP1 by LT might increase this activity (46). Accordingly, a C-terminal fragment of NLRP1b containing the CARD and 56 adjacent amino acids is sufficient for caspase-1 activation with the 56 adjacent amino acids being required for oligomerization (47). However, LTx-mediated cleavage of NLRP1b is still controversial, since another study failed to observe LTx-mediated cleavage of NLRP1b, although LTx was required for autoproteolysis (44).

**Figure 2:**
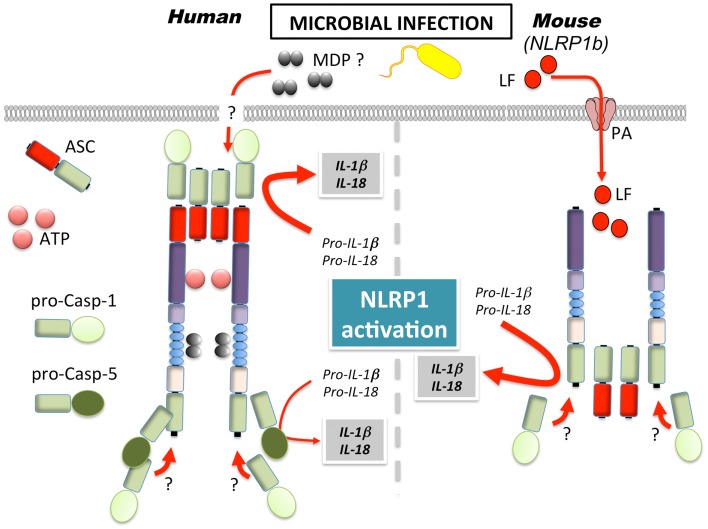
**Mechanism of NLRP1 activation in human and mice**. MDP, muramyl dipeptide; LF, lethal factor; PA, protective antigen.

A first glimpse into the functional importance of NLRP1 was discovered, when genetic mapping identified NLRP1b as the gene responsible for LTx sensitivity in mice. In mice, only NLRP1b, and none of the two other paralogs (NLRP1a and NLRP1c), confer susceptibility to LTx (Figure 2) (48). The exact role of LTx in this context during *B. anthracis* infection is, however, a matter of controversy, since *in vitro* cell death and *in vivo* end-stage LTx-induced disease and death appear to not be linked (49). Furthermore, different mechanisms have been reported for LTx and spores, with the latter promoting an inflammasome response in LTx susceptible and resistant macrophages (50). A similar protective response has also been reported in response to *Toxoplasma gondii* infection, where NLRP1b activation ensured selective elimination of the niche for pathogen proliferation, cytokine release, and effective spreading of danger signals to neighboring cells (51).

Several studies observed NLRP1/NLRP1b sensing of MDP (22, 25, 52). However, while the recently generated NLRP1b deficient mice demonstrated impaired inflammasome response to LTx, the response to MDP was intact and rather NLRP3-dependent (53). Furthermore, NLRP1b has been suggested to sense energy stress in fibroblasts, as a consequence of starvation (54). In particular, NLRP1b senses the reduction of intracellular ATP levels and the subsequent activation of the AMP-activated protein kinase (AMPK). Congruently, a mutation of the ATP binding pocket within the NACHT of NLRP1b yielded a constitutively active inflammasome, suggesting that ATP binding might inhibit, rather than activate NLRP1b, in contrast to what has been reported for human NLRP1 (25, 55).

Underlining its functional importance, further control mechanisms besides RNA splicing may regulate the activity of the NLRP1 inflammasome. The anti-apoptotic proteins Bcl-2 and Bcl-X_L_ were reported to specifically inhibit NLRP1 activation by blocking ATP binding (52, 55). Both proteins appear to bind to the LRR of NLRP1 with their loop region, suggesting that different domains are responsible for their NLRP1 inflammasome-suppressing activity compared to their apoptosis-suppressing activity. Furthermore, recent evidence suggests that NLRP1 may provide a more effective immune response by associating with NOD2 (22). Finally, there is evidence that the anti-inflammatory omega-3 (ω-3) polyunsaturated fatty acids attenuate NLRP1b through interaction of NLRP1b with β-arrestin-2, the downstream scaffold for GPR120 and GPR40 (56).

### NLRP2

Although NLRP2 (Figure 1A), also known as PYPAF2, NALP2, PAN1, and CLR 19.9 failed to affect activation of NF-κB or caspase-1 in initial *in vitro* studies (57), it was later shown to inhibit cytokine-induced NF-κB activation. Subsequently, it was shown that PYD-mediated interaction of NLRP2 with ASC resulted in the abrogation of the expression of NF-κB target genes in the monocytic THP-1 cell line (58). Highly expressed in T-cells, NLRP2 was also found to inhibit NFAT and AP-1, in addition to NF-κB, following TCR activation by anti-CD3 and anti-CD28 antibodies or PMA/ionomycin (59). Besides its transcriptional regulation, biochemical studies in THP-1 cells, suggesting that NLRP2 could assemble into an ASC and caspase-1-containing inflammasome (41). NLRP2 does not contain a FIIND domain, but CARD8 (also known as Cardinal and TUCAN), which is the only other FIIND domain-containing protein besides NLRP1, is recruited to NLRP2 via its NACHT (41). In a manner similar to NLRP1, the FIIND domain of CARD8 is also autoproteolytically cleaved, potentially to promote downstream signaling (43). The *in vivo* function of CARD8 and its role in inflammasome activation, however, is still poorly defined, since CARD8 is does not exist in mice (60). NLRP2 is highly expressed in human astrocytes within the central nervous system and, similar to NLRP3, appears to assemble in an ASC- and caspase-1-containing inflammasome in response to exogenous ATP, as shown by gene silencing (61). In this context, NLRP2 may directly interact with the P2X_7_R and pannexin-1, suggesting a direct effect on the NLRP2 inflammasome, rather than the indirect effect that is observed for NLRP3. However, these findings will need further corroboration, in particular *in vivo*.

### NLRP3

**Figure 3:**
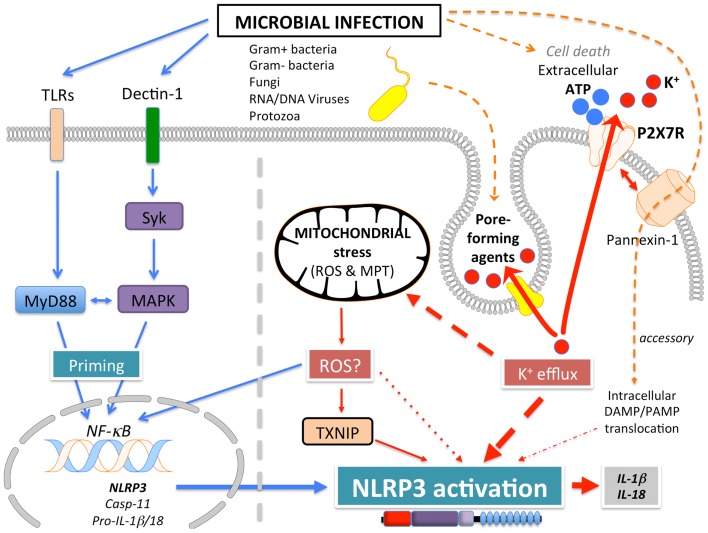
**Mechanism of NLRP3 activation in response to pathogen infection**.

**Figure 4:**
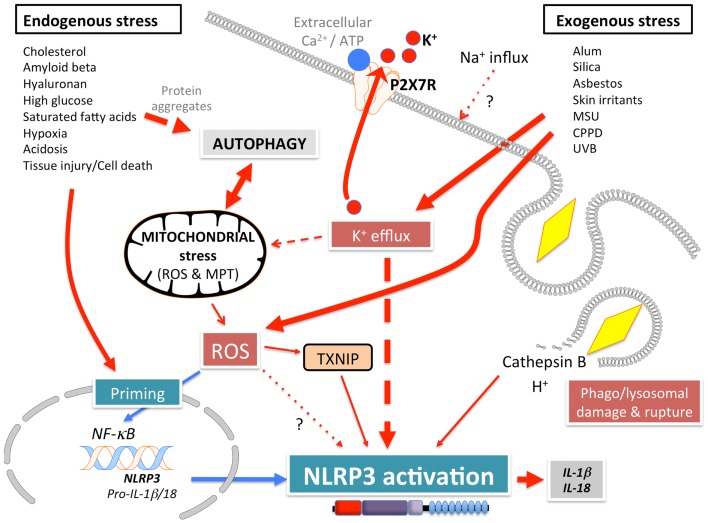
**Mechanism of NLRP3 activation in response to endogenous and exogenous danger signals**.

NLRP3 (Figure 1A), also known as Cryopyrin, NALP3, PYPAF1, CIAS1, CLR1.1, is the best-studied member of the NLRP family. It was initially discovered by positional cloning in the search for the genetic cause of a group of auto-inflammatory diseases, now referred to as Cryopyrinopathies or Cryopyrin-associated periodic syndromes (CAPS) (62). While initial overexpression studies suggested that NLRP3 affects NF-κB activation, NLRP3-deficient mice displayed defects restricted to inflammasome activation (63–66). In contrast to other Nod-like receptors (NLRs), NLRP3 is activated by, and responds to a diverse set of stimuli originating from microbes pathogen-associated molecular patterns (PAMPs) (Figure 3) or from environmental and endogenous danger signals danger-associated molecular patterns (DAMPs), which can be of either soluble or particulate matter (Figure 4). Microbial activators include various Gram-positive and -negative bacteria (*Listeria monocytogenes, Staphylococcus aureus, Vibrio cholera, Neisseria gonorrhoeae*, and others) (64, 67–71), fungi (*Candida albicans, Saccharomyces cerevisiae*) (72), RNA and DNA viruses (adenovirus, influenza virus, Sendai virus, MCMV) (73–75), as well as protozoa (*Plasmodium malariae*) (76–78). The fact that NLRP3 also senses sterile environmental and endogenous stress signals, and promotes inflammatory responses further expands the repertoire of NLRP3 reactivity. Environmental triggers include the particulates alum (79–83), asbestos (84, 85), silica (81, 84, 85), skin irritants (trinitrochlorobenzene, trinitrophenylchloride, and dinitrofluorobenzene) (66, 86), and even UVB radiation (87). An increasing complexity of endogenous danger signals is now also known to activate NLRP3, since the discovery that monosodium urate crystals (MSU) and pyrophosphate dihydrate (CPPD) crystals are able to activate NLRP3 (65). Other known NLRP3-inducing crystals are cholesterol, amyloid deposits (88, 89), hydroxyapatite crystals (90), and hyaluronan (91). In addition to these crystalline danger signals, NLRP3 also senses non-crystalline stress signals, including ATP (64), high glucose (92), and saturated fatty acids (93). The mechanism that causes NLRP3 activation in response to so many different stimuli is still controversial and more discussed below.

#### Basic concepts of NLRP3 inflammasome activation

Based on the diverse structural nature of NLRP3 agonists, the current model assumes that intermediate factors may be involved in sensing of these activators, rather than a direct receptor-ligand interaction. Among all NLRPs, an essential *in vivo* function of the LRR in NLRP activation has only been shown for NLRP3. In contrast to many *in vitro* studies showing that deletion of the LRR renders the NLRP constitutively active, likely because of a lack of autoinhibition, the absence of the LRR *in vivo* renders NLRP3 unresponsive to MSU and ameliorates MSU-induced inflammation in mice (19). Activation of NLRP3 does not fit into a unifying model (94), but it is well-established that NLRP3 activation employs a two-step mechanism.

*Signal 1*: activation of NLRP3, especially in mouse myeloid cells, requires a “priming” step. While it was initially believed that this step is necessary for providing the cytokine substrates, in particular proIL-1β, which is highly inducible by NF-κB, it was subsequently proposed that induction of NF-κB is necessary for transcription of NLRP3 itself (95, 96). This proposal was based on the observation that ectopic expression of NLRP3 uncouples NLRP3 activation from priming (95). In addition to NLRP3 expression, priming has been shown to potentiate NLRP3-specific activation of caspase-1 at short time points that do not affect NLRP3 expression levels and furthermore, also potentiates NLRP3 inflammasome activity following ectopic NLRP3 expression (97). The mechanism behind this observation is likely TLR4-MyD88-dependent deubiquitination of NLRP3 by BRCC3, which is essential for its activation (98–100).

*Signal 2*: subsequently, a specific activating step (signal 2) triggers NLRP3 activation and assembly of the NLRP3 inflammasome. Three main activating mechanisms have been proposed: (1) K^+^ efflux, (2) mitochondrial dysfunction and generation of mitochondria-derived reactive-oxygen species (ROS), and (3) phagolysosomal destabilization in response to particulates (Figures 3 and 4).

(1) ATP is released into the extracellular space after tissue injury and cell death. The extracellular ATP then triggers the purogenic P2X_7_R, which is an ATP-gated K^+^ ion channel, that facilitates K^+^ efflux, which activates the NLRP3 inflammasome (64, 101, 102). Although the interaction of P2X_7_R with the hemichannel protein pannexin-1 was initially proposed to allow influx of PAMPs/DAMPs into the cytosol through a 900 kDa pore, based on pannexin-1 blocking peptides (103). However, this scenario is not any longer considered to play any role in NLRP3 activation, since pannexin-1-deficient macrophages exhibit no defect in NLRP3 activation (104). Similarly, microbial pore-forming toxins (such as hemolysins) on the cell surface or on phagolysosomal membranes trigger K^+^ efflux and NLRP3 activation (105). The precise mechanism by which low K^+^ levels affect NLRP3 activation is not understood. While K^+^ efflux in NLRP3 activation is well-established, Ca^2+^ mobilization and Ca^2+^-mediated signaling has also been linked to NLRP3 activation, but this is controversial (75, 106–108). ATP induced Ca^2+^ signaling is regulated by the calcium-sensing receptor (CASR), phospholipase C-mediated generation of inositol-1,4,5-trisphosphate, IP3R mediated release of Ca^2+^ from the ER, and store-operated Ca^2+^ entry (SOCE) mediated influx of extracellular Ca^2+^, which is important for NLRP3 inflammasome activation by extracellular ATP. Hence, caspase-1 and IL-1β processing and release are also controlled by PLC, IP3R, and SOCE (75, 106–108). In addition to ER stores, Ca^2+^ influx has also been proposed to occur through the plasma membrane channel TRPM2 (108). However, the involvement of Ca^2+^ in NLRP3 activation has been recently disputed and linked to the precipitation of insoluble particulates, which then activates NLRP3 in a K^+^ efflux-dependent manner (102).
(2) A second mechanism proposed to contribute to NLRP3 activation, involves mitochondria and generation of ROS (92, 109, 110). However, involvement of mitochondria and mitochondria-derived molecules, including mROS in NLRP3 inflammasome activation is controversial with arguments found for and against throughout the literature. ATP-mediated ROS production is necessary for caspase-1 activation (111) and initial studies linked NADPH oxidase-produced ROS to NLRP3 activation (76, 85). Interaction of NLRP3 with the thioredoxin (TRX)-interacting protein TXNIP through its LRR, has been proposed as a mechanism, since NLRP3 agonists caused ROS-dependent dissociation of TXNIP from TRX (92). However, subsequent studies in chronic granulomatous disease (CGD) patients disproved these earlier observations. CGD patients lack p22^phox^, which is essential for the proper function of the NADPH oxidase Nox1-4, but CGD macrophages showed either no defect in IL-1β release (112), or even an increased caspase-1 activity and IL-1β release (113, 114). This is in agreement with the finding that ROS actually inhibit caspase-1 through reversible oxidation and glutathionylation of two redox-sensitive cysteine residues (C^397^ and C^362^), which is in contrast to an earlier study. Furthermore, the crystal structure of the NLRP3 PYD revealed that it is unique in containing a disulfide bond between C^8^ and C^108^, which could be important for redox potential-dependent regulation (13). Mitochondria are the other main source for ROS, and mitochondria have been linked to NLRP3 activation through mROS generation and as a platform for inflammasome assembly. While mROS are necessary for homeostasis, cellular stress including hypoxia, acidosis, changes in intracellular ionic milieu and membrane damage are known to promote release of mROS (115, 116). It has also been proposed that all NLRP3-activating stimuli induce apoptosis in target cells, thereby causing opening of the voltage dependent anion channel (VDAC), decreases the mitochondrial membrane potential (ΔΨ), generation of mROS, which in turn promotes mitochondrial permeability transition (MPT) and cytosolic release of mitochondrial DNA leading to NRLP3 activation (92, 110, 117). Accordingly, inhibiting VDAC1 and 2, but not VDAC3 decreased NLRP3 activation (110). Furthermore, defect mitophagy or autophagy, and consequently, accumulation of damaged mitochondria, causes NLRP3 activation and elevated IL-1β levels (109, 110, 118, 119). However, autophagy is also involved in degrading ubiquitinated inflammasomes through recruiting the autophagic adaptor p62 (119). Moreover, it has also been proposed that mitochondrial damage does not contribute to NLRP3 activation, but can occur in response to NLRP3-activating stimuli at later time points (102). Additional support for a significance of mitochondria as a platform facilitating NLRP3 activation is supported by studies showing that ER-localized NLRP3 is redistributed to mitochondria upon activation (110). This transport has been shown to occur by a dynein-mediated movement of mitochondria in response to reduced NAD^+^ levels caused by defect mitochondria. This facilitates inactivation of sirtuin 2, an NAD^+^-dependent α-tubulin deacetylase, and consequently, accumulation of acetylated α-tubulin necessary for mitochondrial movement (120). However, mitochondrial ASC and NLRP3 localization is also controversial. Yet another study proposed that the CARD-containing RLR adaptor MAVS is necessary for full NLRP3 inflammasome activation through targeting NLRP3 to mitochondria, which requires a short peptide within the PYD (121). However, MAVS appears to be only necessary for non-crystalline activators, suggesting that other adaptors might be involved in crystalline responses. However, this finding is controversial and has only been partially reproduced in the context of Sendai virus infection (122).
  Altogether, there is widely conflicting information of the involvement of mitochondria and mROS to NLRP3 activation. Analyses of various mitochondria-targeted drugs suggested an involvement of mitochondria and mROS dependent and independent mechanisms (123). But a recent study suggested that, rather than acting on the signal 2 of NLRP3 inflammasome activation, ROS might only be necessary for inflammasome priming through NF-κ activation or deubiquitination (95, 98). Yet, these studies have also been disputed and attributed to the use of high concentrations of ROS inhibitors and proposed that ROS do not play any role in signal 1 and 2 (102).
(3) Reactive-oxygen species are also generated upon lysosomal rupture and leakage of lysosomal contents in the cytosol, as a consequence from the digestion of particulate matter or infection. Phagolysosomal destabilization itself, rather than the absorbed particulate matter, seems to be perceived as the danger signal leading to NLRP3 activation (81, 89). Abnormal release of H^+^ into the cytosol, either from lysosomal rupture or from the activation of a proton-selective ion channel, such as the M2 channel upon infection with Influenza virus (124), activates NLRP3. The lysosomal-derived protease cathepsin B is one of the lysosomal factors that activate NLRP3 (81, 89). However, this finding was dependent on a chemical cathepsin B inhibitor, while cathepsin B^−/−^ macrophages do not show defects in caspase-1 activation (76), suggesting off target effects of this inhibitor (125).

A recent study aimed to provide an explanation for these diverse NLRP3-activating mechanisms, by essentially demonstrating that all tested NLRP3-activating stimuli act through promoting K^+^ efflux and subsequent Na^+^ influx, and that K^+^-free medium alone is sufficient to activate NLRP3 in the absence of any other agonist (102). This study further suggested that neither mitochondrial perturbation nor the generation of ROS directly contributes to NLRP3 activation (102).

#### Special considerations for NLRP3 inflammasome activation and alternative upstream pathways

Several co-factors have been proposed to affect NLRP3 activation in response to all or select stimuli, which, however, in some cases are not as well-established. According to the universal NLR model, NLRP3 likely exists in an inactive, auto-inhibited conformation, which is aided by the interaction with the ubiquitin ligase SGT1 and the heat shock chaperon HSP90 (126). This is in agreement with the above described finding that deubiquitination of NLRP3 is essential for its activation (98–100). Yet another mechanism to maintain an inactive conformation or to prevent oligomerization, has been proposed to be interaction with cAMP via its NACHT. Ca^2+^ signaling through CASR during NLRP3 activation then causes depletion of intracellular cAMP levels and promotes NLRP3 activation (106). Yet another player regulating NLRP3 inflammasome activation, is the double-stranded RNA-dependent protein kinase (PKR), which phosphorylates NLRP3, but also interacts with other NLRs and ALRs (127). Once activated, oligomerization via its NACHT domain also requires ATPase activity and ATP hydrolysis (24). NLRP3 oligomerization is necessary for ASC clustering, which, however, in response to non-crystalline stimuli, may require PYD-mediated interaction with tetrameric guanylate binding protein 5 (GBP5) to facilitate oligomerization (128). Activation of NLRP3 is also inhibited by anti-inflammatory ω-3 polyunsaturated fatty acids through binding of the downstream scaffold β-arrestin-2, as also shown for NLRP1 (56). Furthermore, LRRFIP2 inhibits NLRP3 inflammasome activation by recruiting the pseudo caspase-1 substrate Flightless-I through NACHT-LRR interaction (129).

Although, NLRP3^−/−^ and ASC^−/−^ mice are less sensitive to LPS-induced shock, this only occurs at lower LPS doses and only provides partial protection (64, 130, 131). Contrary, caspase-11^−/−^ mice are fully protected from LPS-induced shock (132). In response to selective Gram-negative *Escherichia coli, Citrobacter rodentium, Salmonella typhimurium*, or *V. cholera*, or upon cytosolic delivery of LPS, caspase-11 is required for full activation of caspase-1 within the NLRP3 inflammasome, which is referred to as the non-canonical inflammasome pathway (132–136). In the presence of NLRP3, ASC and caspase-1, caspase-11 favors secretion of the pro-inflammatory cytokines IL-1β and IL-18. However, in their absence, caspase-11 drives pyroptosis, IL-1α, and HMGB1 secretion. In particular, caspase-11 activation upon infection by *Salmonella* renders cells more susceptible to pyroptosis, which is even detrimental to the host in the absence of caspase-1 (136). Similar to NLRP3, a priming step is necessary to up-regulate caspase-11 transcripts. A TRIF-type I IFN-dependent transcriptional response has been initially proposed (135, 136). However, subsequent studies disputed a TRIF-specific mechanism, but nevertheless highlighted the necessity for TLR-mediated priming to up-regulate caspase-11 (137, 138). However, the LPS sensor upstream of caspase-11, however, is still elusive.

### NLRP4

The function of NLRP4 (also known as NALP4, PAN2, PYPAF4, RNH2, and CLR19.5) (Figure 1A) in innate immunity is still poorly understood. It has not been linked to inflammasome activation, but overexpression studies indicated that NLRP4 modulates NF-κB activation in response to pro-inflammatory cytokines, including TNFα and IL-1β (139). Recently, NLRP4 has been proposed to modulate type I IFN signaling and autophagy, based on gene silencing and overexpression (140, 141). In response to Group A *Streptococcus* (GAS) infection, NLRP4 inhibits the initiation of autophagy through interaction with beclin-1. Interestingly, all other tested NLRs, including NLRC4, NLRP3, and NLRP10 also interacted with beclin-1, potentially indicating this is a more common mechanism of NLRs (140). NLRP4 further interacted with the class C vacuolar protein-sorting complex to inhibit phagolysosomal maturation (140), suggesting that NLRP4 and possibly other NLR family members play a role in autophagosome maturation following bacterial infection. Yet, during viral infection, NLRP4 has been proposed to play a regulatory role within the type 1 IFN pathway in response to dsDNA and dsRNA (141). NLRP4 targets the central type IFN signaling component TBK1 for K48-linked polyubiquitination and degradation, through recruiting the E3 ubiquitin ligase DTX4 to TBK1, resulting in loss of IRF-3 activity. Only the NACHT of NLRP4 is required for this activity. While humans encode only NLRP4, mice encode seven paralog genes, NLRP4a-g, with at least NLRP4b and NLRP4g also inhibiting type I IFN production (141).

### NLRP6

**Figure 5:**
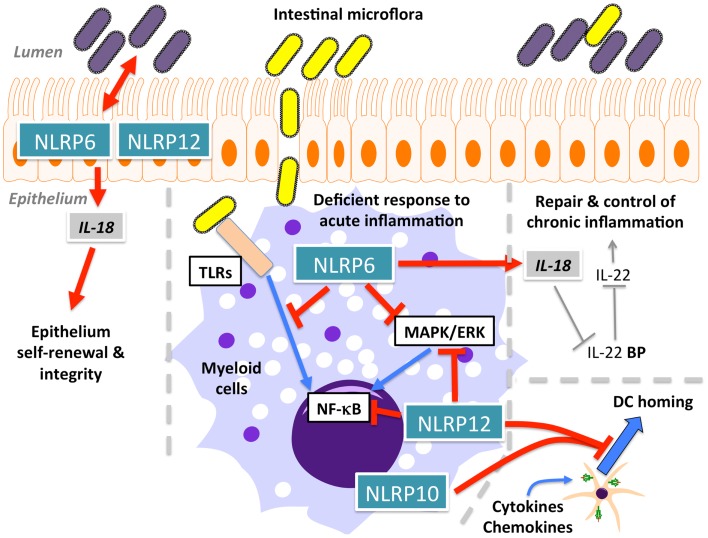
**Function of NLRP6, NLRP10, and NLRP12 in intestinal homeostasis and dendritic cell (DC) homing**.

Initial overexpression studies suggested that NLRP6 (also known as NALP6, PAN3, PYPAF5, CLR11.4) (Figure 1A) mediates activation of NF-κB and caspase-1 in the presence of ASC (57). A subsequent study hinted at a function of NLRP6 within the intestinal epithelium, based on transcriptional profiling (142), and it is now evident that NLRP6 might function differently in myeloid cells and in intestinal epithelial cells. Three recent studies in NLRP6-deficient mice confirmed a role for NLRP6 in the regulation of intestinal host-microbiota (Figure 5) (143–145). NLRP6-deficient mice develop an increased sensitivity to DSS-induced colitis and colitis-induced tumorigenesis, suggesting a protective role of NLRP6 against intestinal inflammation and inflammation-induced cancer (143, 145). Although, it was previously suggested that NLRP6 is mostly expressed in the non-hematopoietic compartment, bone marrow chimera demonstrated the requirement of hematopoietic cells for this function (143). These studies further elute to a function of NLRP6 in intestinal epithelium self-renewal during steady state and during repair after inflammation through suppressing inflammation and associated colorectal carcinogenesis (143, 145, 146). NLRP6 is essential in regulating the interplay between host and gut microflora. Mice deficient in the NLRP6, or a potential NLRP6 inflammasome, although the latter is only based on overexpression data, develop a transferable colitogenic intestinal microbiota due to failure to produce IL-18, a necessary cytokine for the restriction of *Prevotellaceae* and *TM7* species in the steady state and upon DSS treatment through induction of CCL5 and IL-6 (144, 146). These results support the idea that NLRP6-driven IL-18 production from the epithelium is the major contributor to prevent the development of the colitogenic phenotype, as opposed to IL-18 secreted from the hematopoietic compartment. IL-18 is at least partially responsible for the down-regulation of IL-22BP during inflammation, allowing IL-22 to improve epithelial cell repair, while IL-22BP increases again at the end of regeneration with the decrease of IL-18 (147). In addition to restricting colitogenic microbiota species, NLRP6 also functions downstream of TLR signaling to dampen anti-microbial host defense. Rather than contributing to elimination of infections, NLRP6 has a deleterious role within the hematopoietic and the non-hematopoietic compartments and, accordingly, NLRP6^−/−^ mice show increased resistance to infection by extracellular *E. coli*, intracellular *L. monocytogenes* and *S. typhimurium*, and display increased circulatory monocytes and neutrophils upon infection (148). Mechanistically, NLRP6 acts as an inhibitor of MAPK and the canonical NF-κB pathway activated by TLR, but not NLR ligation (148). A potential explanation could be that the full extent of the immune response is required to defend against systemic infection, whereas a more controlled immune response might be required in the case of localized inflammation in the gut. Thus, NLRP6 may play a regulatory role in both scenarios by providing protection against chronic inflammation, but consequently being deleterious during acute infections.

### NLRP7

**Figure 6:**
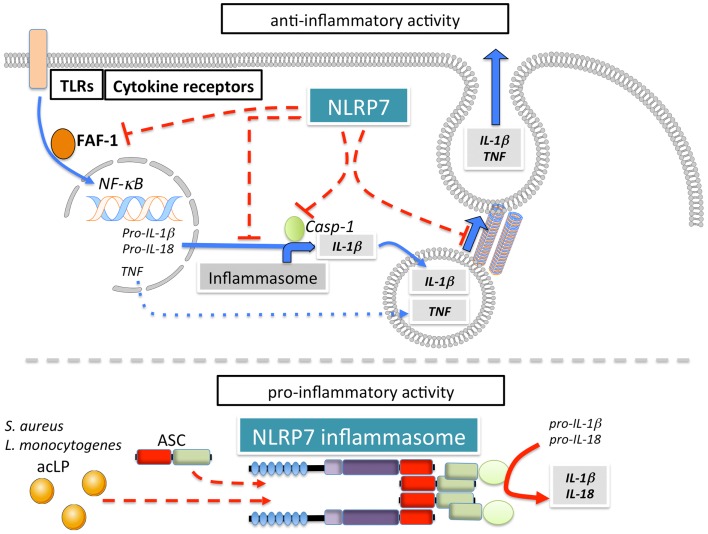
**Pro- and anti-inflammatory mechanisms of NLRP7**.

NLRP7 (also known as NALP7, PAN7, PYPAF3, NOD12, CLR19.4, HYDM) (Figure 1A) is one of four NLRPs, which exist in humans, but not in mice. Although, earlier overexpression studies NLRP7 failed to observe effects on NF-κB and caspase-1 activation (57), several studies since then reported modulation of these pathways by NLRP7. However, conflicting reports describe NLRP7 as either an activator or inhibitor of caspase-1 (Figure 6). NLRP7 has been proposed to directly interact with pro-caspase-1 and pro-IL-1β, without affecting NF-κB (149). It was also proposed that NLRP7 affects secretion of IL-1β and TNFα in PBMCs isolated from patients harboring NLRP7 mutations, which affected its localization to the microtubule-organizing center and the Golgi apparatus, and was associated with a down-regulation of intracellular pro- and mature IL-1β (150). NLRP7 also interacts with FAF-1, which also interacts with several other NLRPs and promotes apoptosis and inhibits NF-κB activation (151). However, modulation of NF-κB was not observed following NLRP7 over expression nor on endogenous level following NLRP7 silencing (57, 71). Overall, there are several potential mechanisms by which NLRP7 could negatively regulate release of inflammatory cytokines (152). In contrast, there is also evidence for a pro-inflammatory role of NLRP7 through the formation of an ASC-containing inflammasome that is triggered in response to bacterial acylated lipoproteins (71). NLRP7 collaborates with NLRP3 and TLR2 in restricting intracellular growth of *S. aureus* and *L. monocytogenes* in human macrophages (71). Similar to NLRP3, NLRP7 also functions downstream of lysosomal damage, with the difference that NLRP7 appears to be only partially sensitive to K^+^ efflux (71). Thus, NLRP7 might contribute to pro- as well as anti-inflammatory processes (152).

### NLRP10

NLRP10 (also known as NALP10, PAN5, NOD8, PYNOD, CLR11.1) (Figure 1A) is the other structurally atypical NLRP besides NLRP1, since it lacks the typical C-terminal LRR. The LRR is essential for NLRP3 activation in response to specific agonists, such as MSU (19), while deletion of the LRR reliefs autoinhibition and renders the NLR active in several *in vitro* studies. Thus, one may predict that NLRP10 might not respond in a stimuli-dependent manner. Over expression studies proposed that NLRP10 oligomerizes with ASC and inhibits ASC-mediated NF-κB activation and apoptosis, as well as caspase-1-dependent IL-1β release (153). Direct caspase-1 inhibition only requires the NACHT domain of NLRP10, but inhibiting ASC-mediated apoptosis, NF-κB and caspase-1 activation required the PYD (154). In contrast to human NLRP10, mouse NLRP10 failed to reduce self-aggregation of ASC, which is required for inflammasome activation. However, transgenic mice ubiquitously expressing high levels of mouse NLRP10 recapitulated the inhibitory effects observed *in vitro*, and mice were more resistant to endotoxic shock *in vivo* (154). In contrast, NLRP10 contributes to host defense to *Shigella flexneri* in epithelial cells and fibroblasts by promoting secretion of IL-6 and IL-8 through induction of NF-κB and p38 signaling pathways, without affecting IL-18 release, arguing against an inflammasome role by gene silencing. This response required the ATPase activity and the PYD of NLRP10 (155). Mechanistically, NLRP10 interacts with signaling components of the Nodosome, including NOD1, RIP2, TAK, and IKKγ in response to *S. flexneri* infection (155). However, NLRP10^−/−^ mice revealed a pronounced defect in mounting adaptive immune responses in the Th1/Th17-dependent experimental autoimmune encephalomyelitis (EAE) mouse model and Th2-dependent OVA- and Alum-driven asthma models (156). These defective Th cell responses were caused by a defective emigration of activated DCs from sites of inflammation to draining lymph nodes, loss of antigen transport, and subsequent priming of CD4^+^ T-cell, though their activation profile remained unaffected (Figure 5). Similar results were obtained in a *C. albicans* dissemination model, in which NLRP10^−/−^ mice displayed increased susceptibility marked by defective Th1 and Th17 responses (157). In both studies, NLRP10^−/−^ macrophages and DCs did not reveal any impact on inflammasome-dependent pathways, and thus above described observations might be caused from overexpression (156, 157). Although hereditary mutations in NLRP3, found in CAPS patients, have been shown to affect Th17 polarization in mice (158, 159), and since CAPS itself is a purely innate immune-driven disease, this is thus the first demonstration of a profound effect of an NLRP on adaptive immunity.

### NLRP12

NLRP12 (also known as NALP12, PYPAF7, RNO2, PAN6, Monarch-1, CLR19.3) (Figure 1A) associates with ASC to form an inflammasome and to promote NF-κB activation, when overexpressed (160). It also enhances expression of the non-classical and classical MHC Class I genes (161). However, NLRP12 also antagonizes signals originating from TLRs and TNF receptor superfamily members upstream of IκBα within the canonical NF-κB signaling pathway by binding to IRAK-1 via its NACHT domain (162) and the non-canonical NF-κB signaling pathway by binding to NIK and inducing its proteasomal degradation (163). Like several other NLRPs, also NLRP12 is an ATPase, and ATP binding/hydrolysis is critical for its function (27). Similar to NLRP3, the interaction of NLRP12 with HSP90 is also important for its stability (164). NLRP12^−/−^ mice recently revealed immune defects. NLRP12 is predominately expressed in DCs and neutrophils, and mice lacking NLRP12 display less severe inflammation in models of contact hypersensitivity (165). However, in contrast to *in vitro* studies, this effect was independent of inflammasome activation and antigen presentation and did not alter inflammatory cytokine levels (165). Similar to NLRP10^−/−^ mice, NLRP12^−/−^ mice also display defects in the migration of peripheral DCs and neutrophils to draining lymph nodes due to an impaired chemokine response (Figure 5) (165). In agreement with *in vitro* data showing that NLRP12 antagonizes NF-κB signaling pathways, NLRP12^−/−^ mice were more susceptible to intestinal inflammation, colitis and the associated colorectal tumorigenesis, due to a failure to resolve pro-inflammatory non-canonical NF-κB, ERK, and AKT signaling, which resulted in elevated levels of pro-inflammatory cytokines and chemokines (Figure 5). Overall, this suggests an important role for NLRP12 in maintaining intestinal homeostasis (166, 167). Although these functions are uncoupled from inflammasome activation, NLRP12 and NLRP3 inflammasomes do contribute to the host defense against *Yersinia pestis* through IL-18 and subsequent IFN-γ production. Surprisingly, NF-κB activation was not affected in this study (168). Thus, dependent on the context and cell type, NLRP12 either promotes or antagonizes immune and inflammatory responses, which has also been observed for several other NLRPs.

## AIM2-Like Receptors

The ALRs AIM2 and IFI16 belong to the PYHIN protein family, which is named after their domain architecture, typically consisting of an N-terminal PYD and one or two C-terminal hematopoietic IFN-inducible nuclear protein with 200-amino acids (HIN-200) domains (Figure 1B). The HIN-200 domain contains partially conserved repeats, which assemble into an oligonucleotide/oligosaccharide-binding fold (OB-fold), which facilitates DNA binding. The OB-fold is a common DNA binding motif, which allows numerous proteins to directly recognize and bind single- and double-stranded DNA (20, 169). While AIM2 preferentially binds dsDNA (170, 171), IFI16 can bind to ssDNA and dsDNA, but only duplex DNA and not the single-stranded form of a Vaccinia virus-derived oligonucleotide was able to induce an IFI16-dependent IFN-β response (35, 172). While only four human *PYHIN* genes exist, this gene cluster is amplified in mice and contains at least 13 predicted and diverse members with only AIM2 being conserved between man and mice (36–38). However, co-localization of several mouse PYHIN proteins with ASC and/or STING, suggests their involvement in inflammasome and/or type I IFN responses (36).

### AIM2

**Figure 7:**
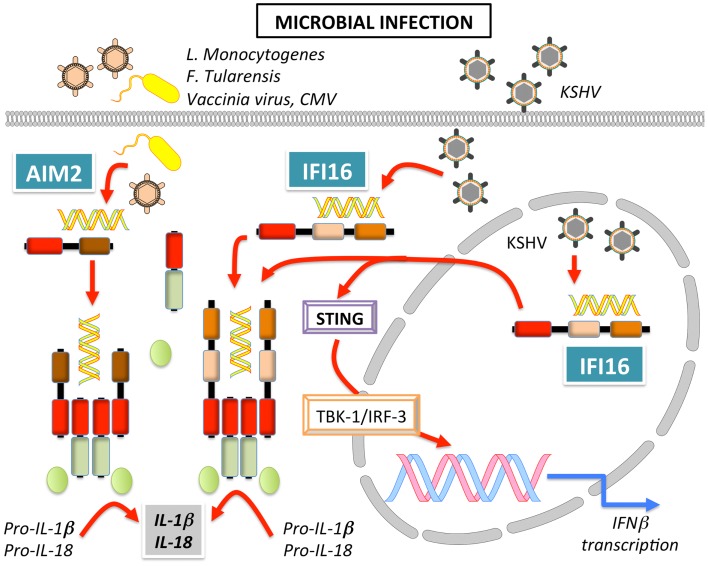
**Signaling of AIM2 and IFI16 leading to inflammasome activation and induction of IFNβ in response to bacterial and viral infection**.

AIM2 or PYHIN4 was initially identified in a human malignant melanoma cell line, where the absence of AIM2 caused increased cell growth and has subsequently been mostly studied in the context of cancer (173). However, a connection between AIM2 and innate immune responses was made when AIM2 was found to recruit ASC to form an inflammasome (Figure 7) (170, 171, 174, 175). The DNA binding capability of the OB-fold within the HIN-200 domain of AIM2 (20) was confirmed to recognize synthetic cytoplasmic dsDNA as well as dsDNA from various pathogens including *Francisella tularensis* (174, 176–178), *L. monocytogenes* (178), Vaccinia virus (170, 174, 178), and MCMV (178), but not DNA from herpes simplex virus type I (HSV-1) and *S. typhimurium* (178). Reminiscent to NLRs, AIM2 activation relies on promoting clustering of ASC and consequently, also caspase-1, but in the case of AIM2, requires the presence of dsDNA (174). Structural analyses proposed that simultaneous binding of multiple AIM2 molecules through electrostatic interaction to the sugar-phosphate backbone of the DNA helix may facilitate the assembly of AIM2 inflammasomes along the DNA staircase (21). *In vivo* experiments also confirmed the importance of AIM2 in host defense, since AIM2^−/−^ mice are unable to limit *F. tularensis* replication, similar to caspase-1^−/−^ mice, and thereby failed to control *F. tularensis* infections (176, 177). AIM2 was also crucial for innate immune responses to MCMV *in vivo*, since the serum levels of IL-18 and the linked production of IFN-γ by NK cells was significantly reduced in the absence of AIM2, which, however, caused an increased splenic virus titer (178). Interestingly, even though cytosolic DNA and some cytosolic bacteria such as *F. tularensis* and *L. monocytogenes* induce an IFN-β response and AIM2 expression is induced by type I IFN, IFN-β signaling is still intact in AIM2^−/−^ macrophages, where it is even enhanced (176, 177, 179, 180). Moreover, type I IFN priming is essential for AIM2-dependent activation of caspase-1, inflammasome-mediated cell death and the release of IL-1β and IL-18 (176, 179). The HIN-200 protein, p202, negatively regulates AIM2 through competition for DNA binding in mice, but due to lacking a PYD, it cannot form an inflammasome (152), but since this protein does not exist in human, alternative regulatory mechanisms may exist. The anti-microbial cathelicidin peptide LL-37 can compete with AIM2 for DNA binding in psoriatic lesions (181).

### IFI16

IFI16 or PYHIN2 was the first human IFN-inducible PYHIN protein identified in myeloid cells (182). Of the three IFI16 isoforms (A, B, and C), the B form is most abundantly expressed (183). IFI16 is also able to bind and recognize DNA to promote transcriptional regulation of genes involved in innate immunity, including type I IFN. Cytosolic DNA recognition promotes recruitment of STING to IFI16 and subsequent NF-κB and TBK-1-dependent IRF-3 activation (Figure 7) (21, 35). Besides this transcriptional response, IFI16 also recruits ASC to form an inflammasome upon recognition of latent viral DNA in the nucleus (34, 172), as well as in the cytoplasm (35, 184) (Figure 7). Curiously, in the steady state, IFI16 localizes mostly to the nucleus, but IFI16 is able to efficiently launch an immune response in the presence of both, nuclear and cytoplasmic DNA. The subcellular localization of IFI16 might determine its function as an IFN-β inducer in the cytoplasm, or an inflammasome-activating PRR in the nucleus. Thus, the immune response following DNA exposure may depend on the cellular or tissue micro-environment, since the function of IFI16 can shift from a transcriptional activator leading to IFN expression to a PRR that causes caspase-1 dependent IL-1β and IL-18 processing in inflammasomes (185). Moreover, one could predict the existence of a regulatory mechanism that restrains IFI16 and AIM2 inflammasome activation in the cytosol upon contact with self-DNA during cell division, since during this process nucleic acids are temporarily exposed to the cytoplasm.

## NLRPs in Inflammatory, Immune, and Metabolic Diseases

As discussed above, PYD-containing PRRs play central roles in key innate immune pathways and are necessary for host defense against a wide range of pathogens and to initiate wound healing of damaged tissue following sterile insults. However, there is now compelling evidence that dysregulated activation of these PRRs, leading to either excessive or impaired activation, causes or contributes to immune-linked diseases. Below we briefly summarize the contribution of NLRPs to auto-inflammatory-, autoimmune-, and metabolic diseases.

### Auto-inflammatory diseases

Auto-inflammatory diseases are generally characterized by recurrent episodes of inflammation and fever in spite of lack of an apparent stimulus and involvement of autoantibodies and autoreactive T cells, causing widespread systemic inflammation which affects multiple tissues and organs (186).

#### NLRP3

Initially a genetic linkage between hereditary point mutations in NLRP3 and auto-inflammatory conditions, now referred to as Cryopyrinopathies or CAPS, was discovered (51). These mutations are gain of function mutations, mostly localizing to the NACHT domain, which create a constitutive active NLRP3 (164). Mutations prevent the inactive conformation of NLRP3 and promote activation in the absence of any specific agonist. Knock-in of CAPS mutations into mice revealed that the disease symptoms are caused primarily by excessive production of IL-1β, but also by pyroptosis in myeloid cells. However, due to IL-1β signaling, mice also show hyperactive Th17 responses (158, 159, 187, 188). Since IL-1β also drives Th17 differentiation in humans (189, 190) it was not surprising that CAPS patients also display significantly increased IL-17 serum levels as well as a higher frequency of Th17 compared to control subjects (191, 192).

Although not driven by hereditary mutations, endogenous crystalline danger signals similarly promote chronic and excessive inflammasome activation and cause crystalline arthropathies and related disorders. Calcium pyrophosphate, monosodium urate, and hydroxyapatite crystal depositions promote NLRP3 activation, excessive inflammation and eventually cause pseudogout, gout, and osteoarthritis (65, 90, 193). Hence, novel treatment regiments with IL-1β blockers have been proven effective (194). NLRP3 is similarly activated following phagocytosis of several other particulate matters. Silica and asbestos fibers activate NLRP3 and result in a non-resolving IL-1β-mediated inflammation, leading to lung fibrosis and ultimately to organ dysfunction in silicosis and asbestosis (84, 85). Cholesterol crystals are also sensed by NLRP3, which contributes to chronic vascular inflammation and ultimately the development of atherosclerosis (195). Similarly, amyloid-β fibrils and islet amyloid polypeptide (IAPP) activate NLRP3, which contributes to Alzheimer’s disease and the progression of type 2 diabetes, respectively (88, 89). Even hemozoin crystals, which are generated during *Plasmodium* infection of red blood cells, trigger NLRP3 activation (76–78), although experimental cerebral malaria progresses independently of NLRP3 (196).

#### NLRP12

In addition to NLRP3, hereditary mutations in NLRP12 have also been linked to auto-inflammatory disease. Guadeloupe fever is clinically similar to CAPS, but is caused by NLRP12 mutations, which truncate the NACHT or delete the LRR (168). However, in contrast to CAPS, anti-IL-1β therapy provided only temporary clinical improvements in two patients, followed by relapse and re-activation of IL-1β secretion, possibly due to enhanced TNFα levels, which were observed in response to the treatment and may have lead to hypersecretion of IL-1β, which circumvented anti-IL-1β therapy (169).

#### NLRP1

Excessive NLRP1-induced IL-1β signaling and pyroptosis can also lead to deleterious organ-specific inflammatory events, such as acute lung injury (53). Moreover, as discussed later, polymorphisms of NLRP1 have been linked to an increased risk developing a number of autoimmune diseases. Although their pathogenesis has not yet been linked to excessive NLRP1 inflammasome activation in humans, it is of interest that analysis of one of these polymorphisms, NLRP1^M1184V^, showed increased NLRP1 autoproteolysis and, consequently, activation of caspase-1 and release of IL-1β (42). Furthermore, *N*-ethyl-*N*-nitrosourea (ENU) mutagenesis screening in mice revealed that NLRP1a^Q593P^, an activating mutation located within the linker connecting the NACHT and LRR, causes lethal systemic neutrophilia, thus linking NLRP1 mutations to hyper-inflammation (197). NLRP1a^Q593P^-driven disease was dependent on IL-1β and caspase-1, but did not require ASC and caspase-11. Moreover, similar to hyperactive NLRP3 mutations, LPS priming of macrophages was sufficient for maturation of IL-1β in NLRP1a^Q593P^ mutant macrophages (197). Interestingly, while the elevated IL-18 release due to NLRP1a^Q593P^ mutation ameliorated the disease, NLRP1a^Q593P^IL18^−/−^ mice displayed increased neutrophilia, independently of IFN-γ, and an accelerated disease onset. IL-18 has emerged as a major intermediate in the crosstalk between the host and commensal microbiota. In this case, the onset and severity of NLRP1a^Q593P^-driven disease was independent, although aggravated, by the presence of commensal microbiota. NLRP1a^Q593P^ specifically caused cell intrinsic hematopoietic stem and progenitor cell defects and particularly manifested in reduced macrophage- and granulocyte-macrophage progenitor cell numbers, caused by pyroptosis, which is only evident in *Il1r*^−/−^ mice in the absence of IL-1β-driven inflammation, and is exaggerated by hematopoietic stress (197). Thus, there is evidence that hereditary mutations in NLRP1 may also lead to excessive inflammasome activation, which is much better understood for NLRP3, as discussed below.

### Autoimmune diseases

Although inflammasome activation is closely linked to innate immune responses, there is now increasing evidence for a role of inflammasomes in adaptive immunity. Although, IL-1β and IL-18 are prototypical cytokines produced by innate immune cells, both are also important for maintaining the Th1-Th17 vs. Th2 balance. Thus, inflammasomes play a role in initiating inflammatory events, but also in the perpetuation of autoimmune diseases characterized by a defect in the T-cell balance.

#### NLRP1

Strong evidence supports an etiologic role of NLRP1 in various autoimmune diseases, since NLRP1 variants have been associated with an increased susceptibility for Addison’s disease, type 1 diabetes, Alzheimer’s disease, celiac disease, Kawasaki disease, autoimmune thyroid disease, generalized vitiligo, systemic sclerosis, and rheumatoid arthritis (198–204). Little is known regarding the mechanism by which NLRP1 mutations affect autoimmunity. However, in generalized vitiligo high-risk NLRP1 haplotypes display elevated IL-1β processing (203), and in rheumatoid arthritis patients, NLRP1 transcripts are elevated (198). Similarly, fibrotic patients display elevated IL-1β levels (205, 206), and systemic sclerosis patients produce considerably higher amounts of extracellular matrix upon exposure to IL-1β (207, 208). This is significant, since caspase-1 is necessary for unconventional protein secretion of numerous leaderless proteins in keratinocytes, which includes several proteins linked to fibrosis in response to UVB irradiation (8).

#### NLRP3

The most direct link of NLRP3 activation to adaptive immunity came from studies with mice harboring CAPS mutations, clearly providing evidence for an abnormal Th1/Th17-skewed immune response (158, 159, 209). Mice displayed spontaneous skin inflammation, consistent with a Th17-skewed response, and produced elevated levels of the Th17-related cytokines IL-17A, IL-21, and IL-22 and the Th17-specific transcription factor RORγt. This is in agreement with an activated phenotype driven by excessive IL-1β levels. In multiple sclerosis (MS), the prototypical Th1- and Th17-derived cytokines, IFNγ, and IL-17, respectively, play an important role. But this concept has been challenged recently by the discovery that only T helper-derived GM-CSF, and neither IFNγ nor IL-17, was essential during the effector phase of EAE, the animal model for MS (210, 211). NLRP3 inflammasome-derived IL-1β is essential for the production of GM-CSF (212), and accordingly, NLRP3 is involved in the pathogenesis of EAE and NLRP3-deficient mice show a strongly ameliorated pathogenesis (213, 214). Nevertheless, this finding is still controversial (215). Also the contribution of NLRP3 to allergic airway disease is still controversial. While some studies observed significantly attenuated airway inflammation, IgE production, and cytokine release in response to OVA in Nlrp3^−/−^ mice (79, 216), others failed to observe any major contribution of NLRP3 (217, 218). Yet another link to adaptive immunity comes from the observation that NLRP3 mediates responses to aluminum hydroxide-containing particular adjuvant formulations (79, 82, 83). However, the precise contribution of NLRP3 to this adaptive immune response is still controversial (80).

#### NLRP10/NLRP12

NLRP10^−/−^ and NLRP12^−/−^ mice both show impaired DC migration to draining lymph nodes, which is independent of inflammasome activation. NLRP12 has been linked to atopic dermatitis and hereditary periodic fever in humans. Hence, NLRP12-deficient mice exhibited attenuated inflammatory responses in mouse models of contact hypersensitivity, which was attributed to a reduced capacity of DC and neutrophil migration and their inability to respond to chemokines *in vitro* (165). Similarly, NLRP10^−/−^ mice displayed a profound impairment in T-cell-mediated immune responses due to the loss of antigen transport to the draining lymph nodes. The defective emigration of DCs from inflamed tissues lead to an almost complete loss of naive CD4^+^ T-cell priming. Hence, there is a critical link between innate immune stimulation, NLRP10 activity, and the immune function of mature DCs (156).

#### AIM2-like receptor

Evidence supports a role of AIM2, IFI16, and the regulatory p202 proteins (p202a and p202b) in the pathogenesis of Sjogren’s syndrome and systemic lupus erythematosus (SLE) (219). In particular, p202 proteins have been linked to increased susceptibility for murine SLE and are regulated by AIM2 (220, 221). However, p202 genes are lacking from human. On the other hand, SLE and Sjogren’s syndrome patients develop autoantibodies to IFI16 in 29 and 70% of all cases, respectively (222, 223), implying a causative link, which is significant due to the reported AIM2 inhibition by IFI16 (224). The most direct evidence shows a contribution of AIM2 to the pathogenesis of Lupus nephritis in an apoptotic lymphocyte DNA-induced SLE model (225). Nevertheless, mechanistic studies implicating ALRs in the pathogenesis of autoimmune disorders are still lacking.

### Metabolic diseases

#### NLRP3

Chronic low-grade metabolic inflammation (metaflammation) is an underlying cause for metabolic diseases. In obesity an excess of nutrients triggers inflammation, since the metabolic surplus induces the expression of inflammatory cytokines, including IL-1β. Hence, there are numerous obesity-related diseases, which include cardiovascular disease, atherosclerosis, insulin resistance, and type 2 diabetes mellitus (T2DM), which are linked to the NLRP3 inflammasome. The NLRP3 inflammasome can be triggered by oligomers of IAPP, which commonly form amyloid deposits in the pancreas during T2DM. In response to IAPP, inflammasome priming, which causes the transcriptional up-regulation of IL-1β, requires a sufficient glucose metabolism and can be facilitated by minimally oxidized low-density lipoprotein (88). Subsequently, IL-1β causes apoptosis of insulin producing β-cells, which results in reduced insulin secretion over time and eventually leads to insulin resistance and T2DM (226, 227). Weight loss in obese individuals with T2DM correlates with reduced NLRP3 expression in adipose tissue. In addition, there is decreased inflammation and improved insulin sensitivity and glucose tolerance in adipose tissue macrophages (ATM) (228). Evidently, the lipotoxicity-associated increase of the intracellular saturated fatty acid palmitate and the metabolite ceramide, are also sensed by NLRP3, in particular following a high fat diet (93, 228). Accordingly, NLRP3^−/−^ mice also show reduced hepatic steatosis and are protected against the accumulation of lipid deposits in the liver (228). Thus, NLRP3 is centrally involved in metabolic health. However, NLRP3, in concert with NLRP6, is also necessary for maintaining a healthy intestinal microbiota to prevent abnormal accumulation of bacterial PAMPs in the hepatic portal circulation.

#### NLRP6

Increasing evidence supports a profound impact of the intestinal microbiota to metabolic health and the intestinal microbiota of obese individuals differs from that of lean people and shows increased prevalence of *Prevotellaceae* (229). NLRP3 and NLRP6 are required for inflammasome-mediated surveillance of the gastrointestinal tract to prevent the spreading of colitogenic microbiota species, including *Prevotellaceae* and *TM7* (144, 230). Restricting these bacteria requires IL-18 and failure promotes CCL5-dependent colonic inflammation and increased TLR4 and TLR9 agonist influx into the portal vein, which eventually causes non-alcoholic fatty liver disease (NAFLD), a comorbidity associated with obesity, metabolic syndrome, and NASH progression (144, 230). Thus, NLRP3 and NLRP6 appear to have a specific protective role within the gastrointestinal tract through production of IL-18, and accordingly, NLRP3^−/−^ and NLRP6^−/−^ mice are more susceptible to colon inflammation and colon cancer (142, 143, 145, 146).

#### NLRP12

Similar to NLRP6, NLRP12 dampens gastrointestinal inflammation and associated tumorigenesis, albeit through a distinct mechanism. Rather than through inflammasome-mediated IL-18 production, NLRP12 prevents intestinal inflammation through dampening NF-κB, ERK, and AKT activation and release of pro-inflammatory cytokines, chemokines, and tumorigenic factors from macrophages and intestinal epithelial cells (166, 167).

## Conclusion

By now, the crucial role of PYD-containing PRRs in host defense is well-established. Although, these PRRs trigger many key innate immune pathways, their contribution to inflammasome activation is currently best understood. Nevertheless, it becomes increasingly recognized that not all PYD-containing PRRs assembly inflammasomes or even promote a pro-inflammatory response. However, the precise signaling mechanisms and in particular, the stimuli that trigger their activation, are largely elusive for most members. The tight affiliation of these PRRs with immune-based diseases further underscores their critical function in maintaining homeostasis, while at the same time opening up exciting avenues for developing novel therapies targeting these PRRs.

## Conflict of Interest Statement

The authors declare that the research was conducted in the absence of any commercial or financial relationships that could be construed as a potential conflict of interest.
